# Evaluation of a push-and-pull strategy using volatiles of host and non-host plants for the management of pear psyllids in organic farming

**DOI:** 10.3389/fpls.2024.1375495

**Published:** 2024-05-22

**Authors:** Bruna Czarnobai De Jorge, Alicia Koßmann, Hans E. Hummel, Jürgen Gross

**Affiliations:** ^1^ Laboratory of Applied Chemical Ecology, Institute for Plant Protection in Fruit Crops and Viticulture, Julius Kühn-Institut, Federal Research Centre for Cultivated Plants, Dossenheim, Germany; ^2^ Laboratory of Plant Chemical Ecology, Technical University of Darmstadt, Darmstadt, Germany; ^3^ Laboratory of Organic Agriculture, Justus-Liebig University of Giessen, Giessen, Germany; ^4^ Laboratory of Biodiversity and Ecological Entomology, Illinois Natural History Survey, Champaign, IL, United States

**Keywords:** repellents, attractive VOCs, essential oils, nanofibers, dispensers, sticky traps, push-and-pull

## Abstract

**Introduction:**

Pear decline (PD) is one of the most devastating diseases of *Pyrus communis* in Europe and North America. It is caused by the pathogen *‘Candidatus* Phytoplasma pyri’ and transmitted by pear psyllids (*Cacopsylla pyri*, *C. pyricola*, and *C. pyrisuga*). Identifying attractant and repellent volatile organic compounds (VOCs) could improve the development of alternative plant protection measurements like push-pull or attract-and-kill strategies against pear psyllids. Our objective was to investigate which chemical cues of the host plant could influence the host-seeking behavior of pear psyllids, and if cedarwood (CWO) and cinnamon bark (CBO) essential oils could serve as repellents.

**Results and discussion:**

Based on the literature, the five most abundant VOCs from pear plants elicited EAG responses in both *C. pyri* and *C. pyrisuga* psyllid species. In Y-olfactometer trials, single compounds were not attractive to *C. pyri*. However, the main compound mixture was attractive to *C. pyri* and *C. pyrisuga* females. CWO and CBO were repellent against *C. pyri*, and when formulated into nanofibers (NF), both were repellent in olfactometer trials. However, CBO nanoformulation was ineffective in masking the odors of pear plants. In a field trial, attractive, repellent CWO and blank formulated NF were inserted in attractive green sticky traps. *C. pyri* captures in traps with CWO NF were statistically lower than in traps with the attractive mixture. Nevertheless, no statistical differences in the numbers of caught specimens were observed between CWO NF and those captured in green traps baited with blank NF. Transparent traps captured fewer psyllids than green ones. In a second field study with a completed different design (push-and-count design), dispensers filled with CBO were distributed within the plantation, and attractive green sticky traps were placed around the plantation. The numbers of trapped pear psyllids increased significantly in the border of the treated plantation, showing that psyllids were repelled by the EOs in the plantation. Although further field evaluation is needed to assess and improve their effectiveness, our results show that these aromatic compounds, repellent or attractive both in nanoformulations and marking pen dispensers, offer great potential as an environmentally sustainable alternative to currently applied methods for managing pear decline vectors.

## 1 Introduction

The pear psyllids (Hemiptera: Psylloidea: *Cacopsylla*) comprise 24 known species of small sap-feeding insects limited in developmental hosts to pear (Rosaceae: *Pyrus*) (Civolani et al., 2023). Several of these species are important pests of commercial pear, most notably *Cacopsylla pyri*(L.) and *Cacopsylla pyricola (Förster)*, in Europe and North America. Pear psyllids cause several types of damage to pear orchards, including russet and downgrading of fruit due to marking of the pear fruit by honeydew and sooty mold, premature leaf drop and tree decline. Most species can transmit the pathogen *‘Candidatus* Phytoplasma pyri’ that causes “pear decline” disease, which causes decreased tree vigor, low productivity, smaller fruit size, and tree dieback. Pear decline is one of the most devastating diseases on *Pyrus communis* in Europe and North America (Seemüller et al., 2011). Along with codling moth [Lepidoptera: Tortricidae: *Cydia pomonella* (L.)], the pear psyllids are the most damaging arthropod pests in commercial pear orchards worldwide (Civolani et al., 2023). Successful phytoplasma transmission experiments with *C. pyricola* (Foerster, 1848) as vectoring species have been carried out in North America (Jensen et al., 1964) and the UK (Davies et al., 1992). The vectoring ability of *C. pyri* (Linné, 1758) was confirmed by transmission tests in France, Italy, and Spain (Lemoine, 1991; Carraro et al., 2001; Garcia-Chapa et al., 2005). In addition, *C. pyrisuga* (Foerster, 1848) has been found to carry the phytoplasma, and successful transmission has been recently reported (Riedle-Bauer et al., 2022). *C. pyri* and *C. pyricola* are polyvoltine [2–8 and 3–5 generations per year, respectively (Civolani et al., 2023)] and can be found on pear trees all year round. In contrast, *C. pyrisuga* is a univoltine migratory species. At the end of winter or in early spring, the adults (remigrants) migrate to their reproduction host plant *Pyrus* spp. where they lay eggs and the immature stages develop. The new generation adults (emigrants) leave their *Pyrus* developmental hosts and spend the rest of the year until the next spring on their overwintering host plants (conifer species) (Ossiannilsson, 1992; Jarausch et al., 2019). According to literature data (Carraro et al., 2001; Riedle-Bauer et al., 2022), a high risk of phytoplasma transmission via *C. pyri* and *C. pyricola* is in late summer to autumn and particularly in late winter and early spring. Due to its low population densities, *C. pyrisuga* is not a primary pest and therefore it is currently not included in pear psyllid control strategies. Its role as phytoplasma vector might have been underestimated so far. Overwintered psyllids likely re-infect trees each spring (Riedle-Bauer et al., 2022).

Discriminating between host and nonhost species progresses through a series of behaviors, which begin with locating a potentially suitable plant, settling and probing, ingesting plant sap, and ovipositing. These behavioral components are regulated by plant-associated cues detected and evaluated by different types of sense organs or sensilla associated with varying structures of insects (Ullman and McLean, 1986; Soroker et al., 2004; Liang et al., 2013; Zhang et al., 2019). Most of these behavioral processes and different sensilla functions are poorly understood. The initial component, locating the host from a distance, is likely governed by visual or olfactory cues detected by ocular organs and olfactory sensilla on the head and antennae (Ullman and McLean, 1986; Soroker et al., 2004; Liang et al., 2013; Zhang et al., 2019). The importance of visual cues is shown by the attractiveness of specific colors to pear psyllids (Adams et al., 1983; Krysan and Horton, 1991; Czarnobai De Jorge et al., 2022; Czarnobai De Jorge et al., 2023). Reflectance peaks that mimic foliar colors (yellow or green hues) were more attractive to pear psyllids than blue, red, or black colors (Adams et al., 1983; Czarnobai De Jorge et al., 2022; Czarnobai De Jorge et al., 2023).

Chemical ecology of pear psyllids has received very little attention despite the major role associated with plant volatiles in host location by pear psyllids. Especially for females, the perception of these odor cues via specialized olfactory receptors is essential to identify suitable plants for feeding and/or oviposition (Mustaparta, 2002; Anton et al., 2007; Cardé and Willis, 2008). Since the volatile blends differ qualitatively and quantitatively between plant species (Bruce et al., 2005; Dötterl et al., 2005; Baldwin et al., 2006), the specific combination of compounds in these blends, many of which are ubiquitous, and their ratios are assumed to drive host plant location in insects (Visser, 1986; Bruce et al., 2005; Tasin et al., 2006).

Taking that into account, untangling the mechanisms of these interactions could eventually provide tools for pest management, improving our understanding of the behavioral responses of insect herbivores to plant volatiles (Rodriguez-Saona and Stelinski, 2009). The manipulation of psyllids’ behavior using volatiles that act as repellent and attractants can be used as an environmentally friendly alternative to control pear psyllids in orchards. Several studies on different species demonstrated that psyllids perceive plant volatiles and evaluated the role of plant chemical cues for host finding (Soroker et al., 2004; Mayer et al., 2008a; Mayer et al., 2008b; Coutinho-Abreu et al., 2014; Rid et al., 2016; Alquézar et al., 2017; Gallinger et al., 2020). Some of them showed that psyllids responded to the odor of several host plants in a Y-tube olfactometer (Mayer et al., 2008b; Wenninger et al., 2009; Patt and Setamou, 2010) and that its antennae detected foliar volatiles in electroantennography tests (Wenninger et al., 2009; Gallinger et al., 2020). Herbivores with multiple generations per year are confronted with substantial variations in the volatile organic compounds (VOCs) emitted by their host plants across a growing season (Tasin et al., 2010; Najar-Rodriguez et al., 2013). One way to deal with variations in plant volatile blends is to respond to a specific set of compounds common to all host plants (Patt and Setamou, 2010). The odor profiles released from pear trees at different phenological stages were rarely studied (Scutareanu et al., 1997; Najar-Rodriguez et al., 2013). Some volatiles were detected constantly over the season (Najar-Rodriguez et al., 2013): Esters: (Z)-3-hexen-1-yl acetate, methyl salicylate, terpenoids: α-pinene, (Z)-ocimene, (β and E)-ocimene and the alcohol (Z)-3-hexen-1-ol. Methyl salicylate, cis-3-hexenyl acetate, ocimene, and cis-3-hexenol were also identified as components of pear leaf fragrance in studies by Scutareanu et al., 1997 (Scutareanu et al., 1997) and Miller et al., 1989 (Miller and Cowles, 1990). However, little information is available on the seasonal dynamics of volatile emissions by pear trees affecting pear psyllids’ behavior.

Reduction of host attraction or masking the host odors is one of the important factors in reducing plant colonization by insects. Essential oils (EOs) are known to affect the behavior of arthropods, serving as a repellent (da Camara et al., 2015) either locally or at particular distances, dissuading an arthropod from landing on a leaf surface with the purpose of oviposition (Benelli, 2015; Ribeiro et al., 2016; Fouad and da Camara, 2017; Lobo et al., 2019), and inhibiting feeding activity (Ali et al., 2017). EO can be used as repellents due to the presence of substances that bind to the proteins of odor receptors (Tyagi et al., 2016), which can lead to diminished feeding on the part of pests and consequent reduction in crop damage (McKenzie et al., 2010). EO of cedar, *Juniperus* genus has been demonstrated to possess bioactivity against several insects, e.g., *Anopheles stephensi*, *Aedes aegypti*, *Culex quinquefasciatus* (Prajapati et al., 2005), *Sitophylus oryzae* (Athanassiou et al., 2013; Dane et al., 2016), *Tribolium castaneum* (Bouzouita et al., 2008; Athanassiou et al., 2013), *Pseudaletia unipuncta* (Rosa et al., 2010), *Xenopsylla cheopis* (Dolan et al., 2014), *Resseliella oculiperda* (van Tol et al., 2007), *Reticulitermes speratus* (Park and Shin, 2005), *Acanthoscelides obtectus* (Papachristos and Stamopoulos, 2002) and the carrot psyllid, *Bactericera cockerelli* (Diaz-Montano and Trumble, 2013). Moreover, literature reports the antimicrobial (Costa et al., 2015; Huerta et al., 2016) and insecticidal (Carvalho et al., 2017; Jeon et al., 2017) activity of cinnamon essential oils from species of the genus *Cinnamomum* spp. However, there are no reports on the repellent activity of the essential oil from *Juniperus mexicana* and *Cinnamomum zeylanicum* against pear psyllids. The essential oil market has had the most substantial growth of all the botanical pesticide markets in recent years. Their widespread use as herbal medicines in Europe, Japan, and North America has increased confidence in their safety.

For the development of alternative plant protection measurements like push-and-pull or attract-and-kill strategies against psyllids, the identification of attractant and repellent VOCs is essential, and the production of dispensers that can reduce the amount of volatiles to be used in field conditions by equilibrating and stabilizing the release rates of such compounds is necessary. Despite the immense potential of EOs for insect pest control, they have disadvantages such as their high cost of production, low vapor pressure, high volatility, low residual effect, pungent odor, and phytotoxicity, properties that limit their applicability (Isman et al., 2011). To overcome these disadvantages, incorporating EOs into a controlled release dispenser can prevent its rapid evaporation and degradation, enhancing stability and maintaining the practical application to its minimum (Ghormade et al., 2011; Czarnobai de Jorge and Gross, 2021). The use of nanofibers as dispensers for kairomones in plant protection for disrupting insect chemical communication offers a novel approach in organic and integrated plant production. Nanoformulations principal advantages are a highly controlled spatiotemporal release rate of volatiles and improved climatic stability (Czarnobai de Jorge and Gross, 2021). In addition, the nanoformulation, compared to pure EOs (i.e., non-formulated), is expected to be more effective against pests and less toxic towards non-target organisms, reducing chemical-synthetic pesticide applications (Devi and Maji, 2011; Anjali et al., 2012).

Thus, this work is undertaken to investigate the electrophysiological and behavioral responses of *C. pyri* and *C. pyrisuga* adults to VOC blends and single compounds emitted by the host, based on already published data (Najar-Rodriguez et al., 2013). Furthermore, we evaluate repellent activities of EOs extracted from *Juniperus mexicana* (cedar wood oil) and *Cinnamomum zeylanicum* (cinnamon bark oil) and their nanoformulations against *C. pyri*, in laboratory. In field experiments we tested selected attractive and repellent volatiles, formulated in biocompatible nanofibers or marker pen dispensers, in attractive sticky traps by counting trapped psyllids.

## 2 Materials and methods

### 2.1 Insects

Adults of *C. pyri* were collected from early spring (March/April) until late summer (August/September), while *C. pyrisuga* adult remigrants (overwintered adults) were caught only in early spring. Insects were sampled from *Pyrus communis* cv. Williams Christ trees at experimental orchard of the Julius Kühn-Institut (JKI) in Dossenheim, Germany. Psyllids were captured using the beating tray method, according to Weintraub and Gross, 2013, and the species were identified using the keys of Burckhardt and Hodkinson, 1986 and Ossiannilsson, 1992. Collected *C. pyrisuga* individuals were reared in a laboratory culture at the JKI (Dossenheim, Germany). They were maintained without exposure to insecticides on healthy potted *P. communis* cv. Williams Christ plants in 47.5 × 47.5 × 93 cm rearing cages (Bug Dorm, NHBS, Devon, UK) in a climatic chamber with 20°C (day) and 15°C (night) temperatures under long-day conditions (L16:D8) and 55% relative humidity (Görg et al., 2021). In olfactometer tests, adult females of *C. pyri* were sampled daily from the experimental orchard, and newly emerged *C. pyrisuga* emigrants were acquired from the lab rearing. Since females play a significant role in population growth, in laboratory trials only female were used because they often choose specific host plants for oviposition and feeding (Mustaparta, 2002; Anton et al., 2007; Cardé and Willis, 2008).

### 2.2 Chemicals

The standard mixture mimicking bioactive post-flowering pear foliage volatiles (Najar-Rodriguez et al., 2013) is shown in **Table 1**. The compounds used are: Ocimene (mixture of isomers, stabilized, ≥90% purity), α-pinene (analytical standard, 98% purity), methyl-salicylate (ReagentPlus^®^, ≥99% purity), (Z)-3-hexenyl acetate (analytical standard, ≥98% purity), (Z)-3-hexene-1-ol (analytical standard, ≥96.0% purity), Furthermore, two EOs, cedar wood oil (CWO, Texas *Juniperus mexicana* Schiede, 100% purity) and cinnamon bark oil (CBO, *Cinnamomum zeylanicum* Blume, 100% purity) used in this study were purchased from Sigma-Aldrich Chemie GmbH, Germany.

**Table 1:** Components of the synthetic volatile blends and single compounds mimicking the pear volatiles.

*Compounds*	*Mass concentration* *(mg/mL)*	*Mx* *1*	*Mx* *2*	*Mx* *3*	*Mx* *4*	*Mx* *5*	*Mx* *6*	*Mx* *7*	*Mx* *8*	*Mx* *9*	*Mx* *10*	*Mx* *11*
*Ocimene*	493.25	X	X		X	X						X
*(Z)-3-hexenyl acetate*	149.44	X		X			X			X		
*Methyl salicylate*	40.27	X	X	X	X				X			
*α-Pinene*	29.50	X	X			X		X				
*(Z)-3-Hexenol*	24.93	X		X			X				X	

### 2.3 Electrophysiological responses of pear psyllids to pear volatile compounds (EAG)

Electroantennographic studies (EAG) were conducted on live psyllids (Wenninger et al., 2009; George et al., 2016; Gallinger et al., 2020) to investigate the olfactory perception of the test compounds mimicking bioactive post-flowering pear foliage volatiles. EAG data were collected on female *C. pyri* and *C. pyrisuga* adults (2–4 weeks old). Psyllids were placed in pipette tips (200 μL) using a small piece of cotton wool. Psyllids were gently pushed through the small end of the plastic pipette tip until the head and antennae were exposed. The pipette tip was threaded to a custom stage that held the psyllid in place before placing electrodes. The psyllid in the pipette tip was mounted 1 cm in front of a filtered and humidified airstream. The air was passed through the antennae with a continuous flow of 1.23 Ln/min. Sharp glass electrodes were prepared using a micro-electrode puller (PN-3, Narishige, Japan; Glass capillaries, 0.58 mm ID, Science Products, Hofheim, Germany). The glass capillary was filled with Ringer solution (NaCl 7.5 g, KCl 0.35 g, CaCl2 0.21 g, 1 L H2O) and mounted on a silver wire electrode holder. With a micromanipulator, the indifferent electrode (prepared likewise mentioned above) was inserted into the insect’s mesonotum, and the distal end of the antenna was placed in the glass capillary connected to the recording electrode (INR-II, Ockenfels Syntech^®^). Tested single volatiles were diluted in methylene chloride (DCM) to concentrations of 1, 10, and 100 µg/µL, and for the mixture, Mx1 dilution series of 1:1000, 1:100; 1:10 and 1 (undiluted) were tested. An aliquot of 10 μL of the dilutions was pipetted on filter paper (Type 413, VWR Collection) and inserted in glass Pasteur pipettes (23 cm). Cartridges were prepared fresh, and the solvent was evaporated for 3 min before puffing over the antenna. The volatile mixtures and individual compounds were applied to the antennae from the smaller to the higher concentration for every replicate (n=10). The stimulus was passed through the antenna for 1 s with a flow of 1.46 Ln/min air puff via the pedal switch connected to the data acquisition controller (IDAC-2, Ockenfels Syntech ^®^). Time intervals of 60 seconds between consecutive puffs were given to allow the antenna to recover. An air puff was applied before each test compound, and a negative control puff (DCM) was used at the beginning and end of each experiment. The reactivity of the antenna was verified by puffing 1000 μg hexanal (10 μL of 100 μg/μL) at the beginning and end of each trial. Proper preparations remained functional for several hours. The antennal responses from ten females of each species were recorded with EAGPro software (version 1.1, Ockenfels Syntech^®^) and extracted for analysis.

### 2.4 Nanofibers production

#### 2.4.1 Preparation of polymer solution and electrospinning

Preparations of polymeric solution and the electrospinning technique were performed as already described by Czarnobai De Jorge et al., 2022. The polymers, Poly-ϵ-caprolacton (PCL, Mw 80 000 g/mol, Sigma Aldrich) and cellulose acetate (CA, Mw 50 000 g/mol, Carl Roth) PCL/CA (1:1) were dissolved in acetic acid (AA, ROTIPURAN^®^ ≥99% purity, LC-MS Grade, Carl Roth) and formic acid (FA, ROTIPURAN^®^ ≥98% purity, p.a., ACS, Carl Roth), AA: FA (1:1) concentration was kept at 15% (w/v) with respect to solvent. The solutions were made by continuously stirring the mixture overnight at 1500 rpm at room temperature to have a homogenous solution. Two formulations were performed (Civolani et al., 2023): for the attractive mixture (Mx1), the amount of the synthetic mixture in the solutions was 0.1, 1, and 5% (v/v) (Seemüller et al., 2011); Repellent EOs, CWO, and CBO were formulated with 10% (v/v).

We used a commercially available electrospinning unit from Linari Engineering, Italy. A 4 ml polymer solution was taken from the stock via a 5 ml syringe fitted with a nozzle of 0.8 mm diameter. The syringe was placed on a pumping machine that provided a controlled flow rate. Fibers were collected on aluminum films, which were placed on the grounded collector at an 11 cm distance from the needle tip (nozzle-to-collector distances) and an applied voltage of 12 kV.

### 2.5 Olfactometer Bioassays

A dynamic Y-shaped olfactometer was used with the following specifications: glass tube, entrance arm length: 12.5 cm, test arm length: 8 cm, inner diameter: 1 cm, angle: 75°, mounted on a board at 40°from the horizontal plane. All experiments were conducted in a dark room with a light source (LED-Lupenleuchte, Purelite, UK) mounted 45 cm (280 lx) above the middle of the olfactometer. The experiments were conducted between 12:00 a.m. and 6:00 p.m. at room temperature (20–26°C and 30–35% RH). A charcoal-filtered and humidified airflow of 40 ml/min (with a max. difference of 1 ml/min) was pumped through the odor source into the test arms. The detailed construction of the olfactometer and procedures of bioassays have been described by Gallinger et al., 2020; Czarnobai De Jorge et al., 2022. Single psyllids were collected in small plastic vials and were kept overnight in the fridge at 6°C. About an hour before the experiment, insects were removed from the refrigerator and kept at room temperature. A single female was introduced at the base of the trunk of the Y-tube olfactometer. Psyllids were observed for 5 min, and the first choice and residence time were noted. Each psyllid was used only once. The number of psyllids that entered one of the test arms (1 cm) and stayed there for at least 30 s was counted. Psyllids that did not reach one of the test arms within 5 min were recorded as “no choice.” After the bioassays, all tubes, valves, and glass olfactometers were cleaned with ethanol (70%) and heated at 230°C (except plastic valves: 60°C) for three hours. After five repetitions, the Y-tube olfactometer and the side on which the treatment was presented were swapped to avoid any positional bias. The following experiments were conducted:

#### 2.5.1 Synthetic blends and single compounds of pear volatiles

Behavioral assays were carried out to determine the responses of (Civolani et al., 2023) wild adult female pear psyllids, *C. pyri*, to synthetic blends and single compounds of pear volatiles (attractive compounds). The reaction of Mx1 on *C. pyrisuga* was also evaluated. However, because of this species’ low number of insects captured, further testing with individual compounds and other blends was impossible. Each arm of the olfactometer contained a piece of filter paper (2.5 x 2.5 cm) with 3 µl of a 1:1000 dilution of synthetic compounds vs. solvent control (DCM) (**Table 1**). To observe if females *C. pyri* could differentiate between the synthetic formulation and the host odors, one fresh twig of potted pear plant was carefully wrapped in oven plastic bags (Toppits, Melitta, Minden, Germany, 31 × 50 cm) and connected to the test arm. In the other arm, a filter paper with 3 µl of a 1:1000 dilution of Mx1 was used and inserted into an empty oven bag. The system was equilibrated for 30 min. To ensure an equal airstream, the flow of each arm was adjusted with plastic valves and controlled by a flowmeter (MASS-STREAM, M + W Instruments, Allershausen, Germany) at the outlet of the oven bags.

#### 2.5.2 Essential oils

The essential oils CWO and CBO were tested by inserting a piece of filter paper (2.5 x 2.5 cm) with 3 µl of the EO in a concentration of 5% vs. solvent control (DCM).

#### 2.5.3 Nanoformulations

Test with nanofibers loaded with the attractive mixture Mx1 was conducted by inserting a 3.0 mg piece of nanofibers in concentrations of 0.1, 1.0, and 5.0% in one olfactometer arm, and as control, only air was pumped in the system. Test with nano-encapsulated essential oils was conducted by inserting a 3 mg peace of nanofibers loaded with 10% oil into the arm of the olfactometer, and as control only air was pumped in the system. To observe the host odor masking effect of essential oil-loaded nanofibers, one fresh twig of pear plant together with a 3 mg-piece of nanofiber containing one of the EOs was carefully wrapped in oven plastic bag as described above and connected to the test arm. The control arm was connected to an empty oven bag where clean air was pumped.

### 2.6 Binary−choice oviposition bioassay

**Figure 1:**
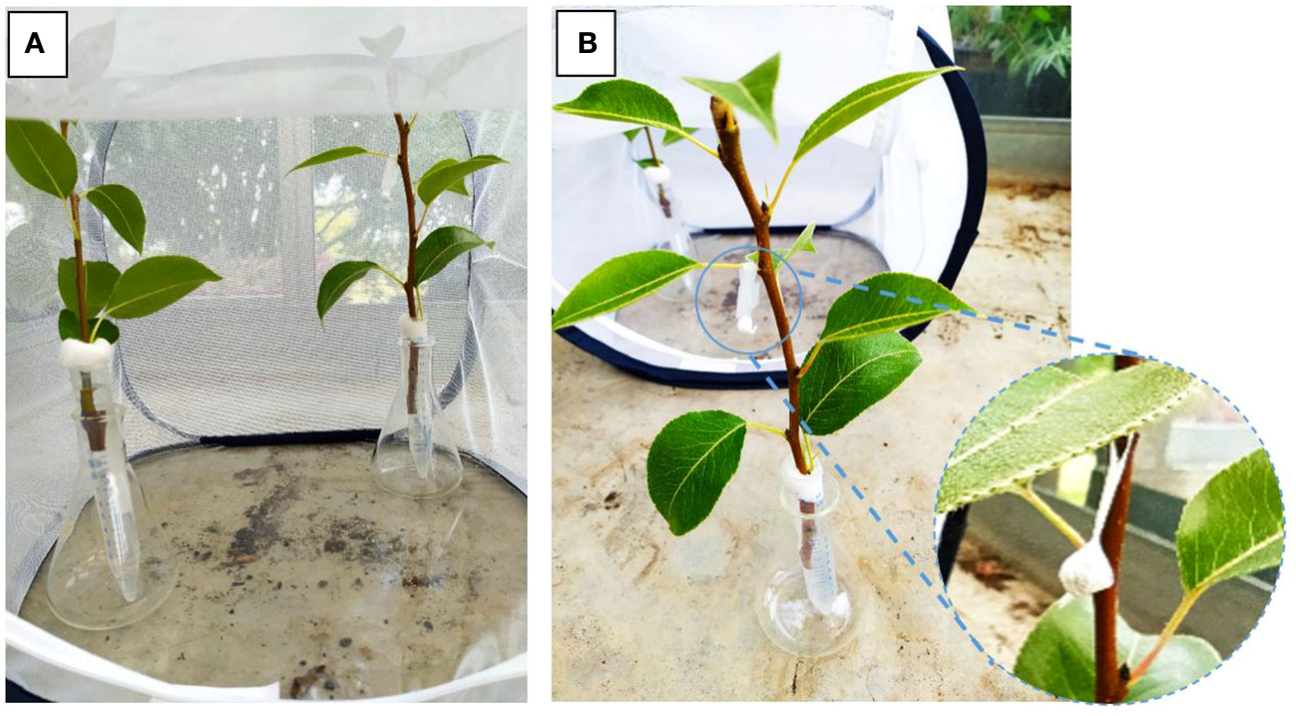
Setup of binary−choice oviposition bioassay on cages: **(A)** disposition of branches on cage (30x30x30cm); **(B)** detail of CWO nanofibers dispenser hanging on leaf.

For conducting binary-choice oviposition bioassay, two pear twigs (20 cm long, 6–8 leaves per twig) were cut from 3-year-old potted pear plants and offered simultaneously to female *C. pyri*. For this, one twig was treated with 30 mg of CWO-formulated nanofiber, and the other remained untreated (**Figure 1**). Pear twigs were inserted into a 15 ml Falcon tube containing water and placed inside 30 x 30 x 30 cm rearing cages. One female was placed in the middle of each cage and left to oviposit for three days (72 h). Afterward, twigs were removed from cages, and the location and number of eggs were assessed with a binocular stereomicroscope (Stemi 508, Carl Zeiss AG, Oberkochen, Germany. The experiment was replicated with 20 females under rearing conditions.

### 2.7 Weight loss investigations of dispensers

Weight loss is an effective parameter in the release behavior of volatiles from dispensers. To evaluate the release rates from nanofibers used in field trials, an amount of 3.0 g of each nanofiber loaded with CWO or Mx1 was weighed once every seven days until 28 days (n=3). Nanofibers were kept under a lab hood under controlled conditions (Temperature 24°C and 30% RU). For the release rates of marking pens, dispensers were hung on trees in the experimental field in May, and the weight was measured after 7 and 14 days under field conditions (n=5).

### 2.8 Field trials

#### 2.8.1 Trial 1: nanofiber dispensers inside traps

Field experiments were conducted to observe if attractive green sticky traps (Krysan and Horton, 1991) equipped with nanofiber formulated with the repellent CWO would reduce the catches of psyllids in comparison with traps equipped with blank nanofiber, and if nanofibers formulated with the attractive Mx1 would yield higher psyllids captures. Therefore, field experiments were conducted in a non-commercial pear orchard in Germany (Julius Kühn-Institut, Dossenheim, Germany). The orchard comprises nine rows (4 m between rows), each containing 53 pear trees (2.5 m between plants). Sticky traps equipped with green-colored transparent films (#068) were used to capture pear psyllids as described by Czarnobai De Jorge et al., 2022; Czarnobai De Jorge et al., 2023. The traps were fabricated from transparent 0.02-mm-thick rigid vinyl plastic cylinders, 9 cm in diameter x 25 cm in length, coated with a thin layer of insect glue, provided by Insect Services GmbH, Berlin, Germany. Traps were designed with 16 holes (1.5 cm diameter) evenly distributed (5 cm between each other).

**Figure 2:**
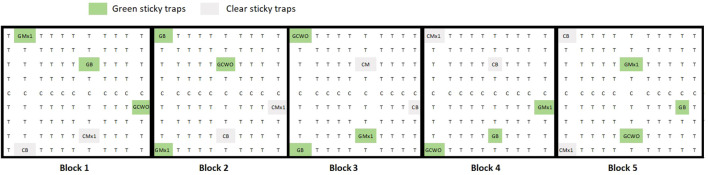
Schematic view of the experimental pear orchard of field trial 1, T = pear trees cv. Williams Christ, C= pear trees cv. Conference. Different trap types or treatments are indicated as follows: GMx1= green traps with nanofibers Mx1 1% loaded, GB= Green traps with blank nanofibers, GCWO = green traps with nanofibers CWO 10% loaded, CMx1= Clear traps with Mx1 1% loaded nanofibers, CB= clear traps with blank nanofibers. Five repetitions of each trap type were distributed over five blocks in the orchard.

The following trap treatments were tested: 1) green traps baited with 3 g of nanofibers with 10% CDR oil (n=5) (Seemüller et al., 2011), green traps with 3 g blank nanofibers (n = 5) (Jensen et al., 1964), green traps with nanofibers with 1% Mx1 (n=5) (Davies et al., 1992), completely transparent (clear) traps baited with 3 g of nanofibers with 1% Mx1 (n=5), or (Carraro et al., 2001) transparent or “clear” traps with 3 g blank nanofibers (n=5). Folded nanofibers were inserted in voile bags (7 x 7 cm) tight with a cord and hung inside the traps as described in Czarnobai De Jorge et al., 2022. We designated five blocks for trap assessments. Within each block, traps were at least 20 m apart. Traps were hung at 1.5 m height and deployed between two pear trees (**Figure 2**). Traps were evaluated weekly for six weeks between June 2021 and August 2021. Captured psyllids were removed from the traps, and the number of pear psyllids trapped was documented. No distinction was made between males and females when evaluating the number of insects caught.

#### 2.8.2 Trial 2: marking pen as repellent dispenser

**Figure 3:**
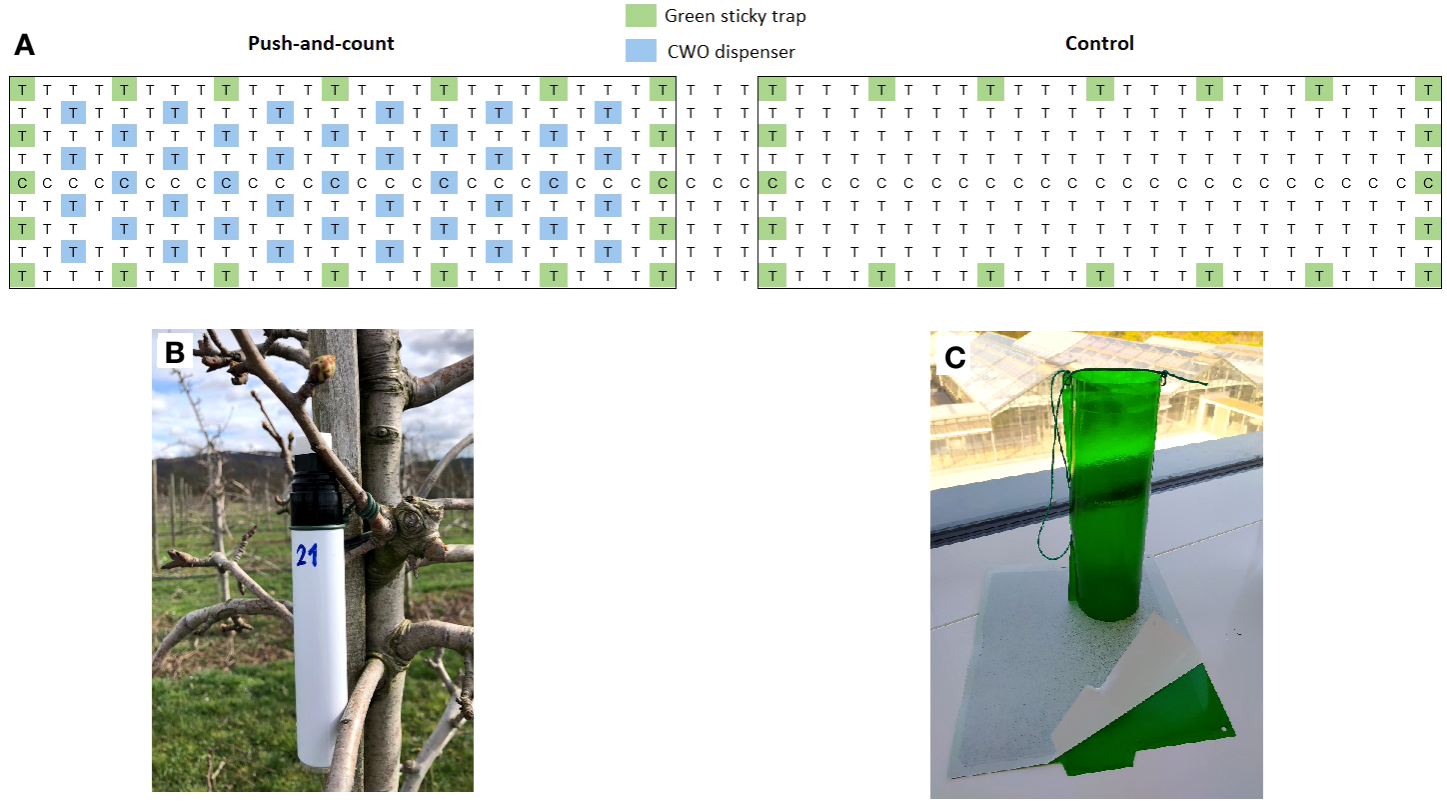
Schematic view of the experimental pear orchard of field trial 2 **(A)**, T = pear trees cv. Williams Christ, C= pear trees cv. Conference. The area was divided in two blocks (treatments): block 1 with marker pen CWO dispensers (“push-and-count”), and block 2 without repellent (control). In the push and count area CWO dispensers **(B)** were hung close to the bark of pear trees, as indicated by blue color. In untreated control, no repellent was applied. **(C)** Green sticky traps position is indicated by green color.

Empty marking pens or felt-tip markers (Edding 850 marker, Edding International GmbH, Ahrensburg, Germany) were used as dispensers to test the so-called “push-and-count” design. The experiment was conducted during the season of 2023 in the same orchard as mentioned in the previous trial. In this experiment, a different setup was proposed. The orchard was divided into two areas; one was treated with the markers filled with 10 ml of CWO, hanging close to the bark of every second tree starting on the third orchard line; the following line was left untreated. This scheme was repeated over the complete treated area. The second area remained untreated as control (**Figure 3**). Commercial attractive green sticky traps with the same wavelength as reported in Czarnobai De Jorge et al., 2022; Czarnobai De Jorge et al., 2023, provided by Insect Services GmbH were hung in the orchard surrounding the border of each site to collect psyllids. One section between treatments was left free to separate the areas. Traps were evaluated periodically between April 2023 and August 2023. Captured psyllids were removed from the traps, and the number of psyllids trapped was documented. As with the previous experiment, an analysis of the number of males and females captured was not carried out.

### 2.9 Statistics

Statistical analysis was performed using the software R (vers. 4.3.2, 2023–10-31 ucrt), “Eye Holes” (Team RC, 2019). This software’s ggplot2 package (Wickham et al., 2020) was also used for graphical representation. Wilcoxon matched-pairs signed-rank test was used to calculate significant differences between EAG responses to the tested volatiles and the control (DCM). In two-choice Y-olfactometer assays, binomial tests were used to determine the significance of choice between treatments odor vs. control. Non-responders were excluded from this analysis. Differences in the number of eggs laid on CWO nanofibers treated and non-treated leaves were evaluated by fitting a generalized linear model with the logarithmized total number of eggs per insect. Due to overdispersion, a negative binomial model with the function was used. For field trials, the averages of psyllids collected by trap were compared using generalized linear models (GLMs), assuming a negative binomial distribution (count data with overdispersion). For the first field trial, we considered ‘week’ and ‘trap type’ as fixed factors in the model. Using a deviation analysis (F-test, link function: ‘log’), we investigated whether the factor’ trap type’ significantly influenced the number of insects oriented toward the traps. In the second field trial, “month” and “treatment” (CWO and untreated) were considered as fixed factors in the model. Interactions between the temporal repetitions of experiments and trap type ‘month: treatment’ were included in the model as fixed factors. The release rate of volatiles from nanofibers was evaluated with the GLM quasi-Poisson model, considering the ‘days’ of exposure of nanofibers and marking pen as a fixed factor. The deviation analysis was performed to evaluate the impact of exposure time ‘days’ on the release rates (F-test, link function: ‘log’). Model tests were performed with a residual diagnostic for hierarchical (multi-level/mixed) regression models ‘DHARMa’ package (Hartig and Hartig, 2017). Multiple pairwise comparisons were calculated with estimated marginal means and 95% confidence intervals with the function ‘emmeans’ from the ‘emmeans’ package (Lenth, 2021) and p values adjustment by Tukey.

## 3 Results

### 3.1 Electrophysiological responses of pear psyllids to pear volatile compounds (EAG)

**Figure 4:**
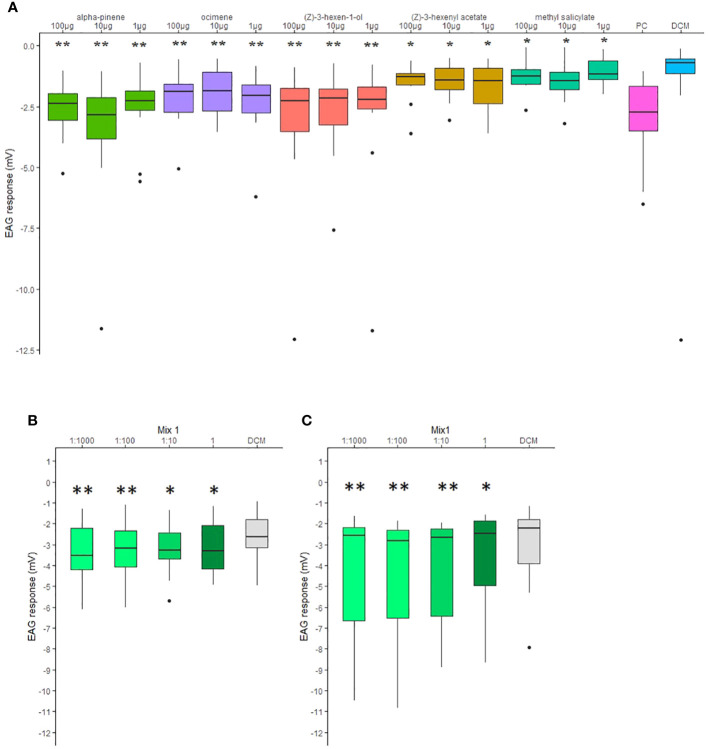
**(A)** Electroantennography (EAG) responses elicited by 100, 10 and 1 µg of synthetic volatiles puffed on the antenna of female *Cacopsylla pyri* (n=10). Hexanal (1 mg) was used as positive control (PC). **(B)** EAG responses elicited by 1:1000, 1:100, 1:10 and 1 dilutions (in DCM) of Mx1 blend puffed on the antenna of female *C pyri*. **(C)** EAG responses elicited by 1:1000, 1:100, 1:10 and 1 dilutions (in DCM) of Mx1 blend on the antenna of female *C pyrisuga*. Statistical analysis was done in comparison to the respective negative controls (DCM) and indicated with *p < 0.05; **p < 0.01 (Wilcoxon matched-pairs signed-rank test). Boxes correspond to the 25th and 75th percentiles, medians are shown as lines, and whiskers extend to 1.5 times of the interquartile ranges.

*C. pyri* female antenna responded to all individual compounds of the attractive mixture (**Figure 4A**). EAG recordings of *C. pyri* and *C. pyrisuga* female, to different concentrations (1:1000, 1:100, 1:10, 1) of the Mix1 were measured to evaluate their olfactory sensitivity. All concentrations elicited significant antennal responses compared to negative DCM controls (**Figures 4B, C**, Wilcoxon matched-pairs signed-rank test, p < 0.05). Both species showed more significant antennal responses to low concentrations of the volatiles mixture 1:1000 and 1:100 compared to 1:10 and 1 (undiluted) (**Figures 4B, C**, Wilcoxon matched-pairs signed-rank test, p < 0.01).

### 3.2 Olfactometer bioassays

#### 3.2.1 Synthetic blends and single compounds of pear volatiles

**Figure 5:**
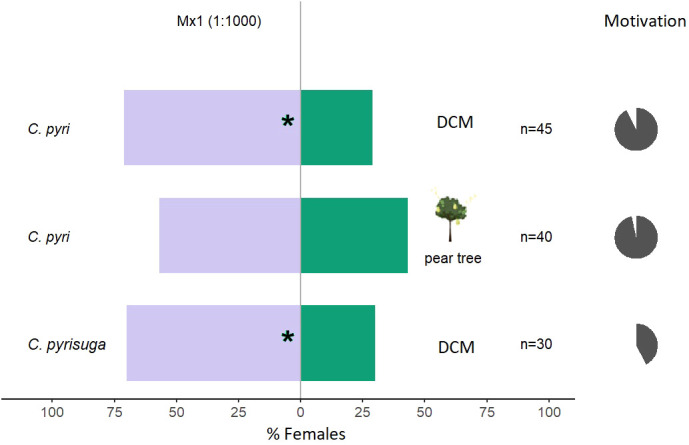
Results of Y-olfactometer-bioassays testing Mx1 blend (**Table 1**) with females of *Cacopsylla pyri* and *C. pyrisuga* against a DCM control or pear tree odor. The letter “n” corresponds to the numbers of insects used for the tests. Results are shown as the percentage of psyllids in each arm. Statistically significant differences are indicated with *p < 0.05 (binomial test). The percentage of psyllids that made a choice (dark gray) or not (light gray) is presented as pie charts on the right (“motivation”).

**Figure 6:**
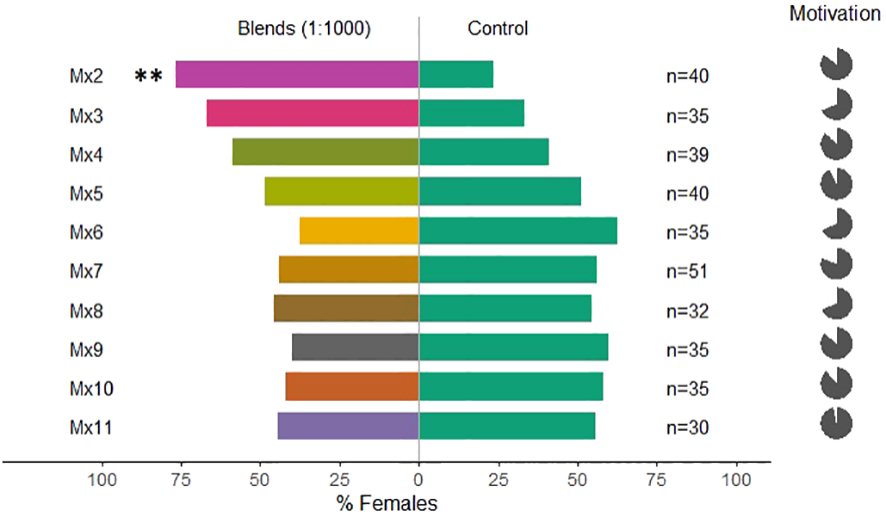
Results of Y-olfactometer-bioassays testing blends and individual compounds (**Table 1**) with females of *Cacopsylla pyri* against a DCM control. The letter “n” corresponds to the numbers of insects used for the tests. Results are shown as the percentage of psyllids in each arm. Statistically significant differences are indicated with **p < 0.01 (binomial test). The percentage of psyllids that made a choice (dark gray) or not (light gray) is presented as pie charts on the right (“motivation”).

Overall, the tested mixtures, only Mx1, with all five compounds, and Mx2 (ocimene, methyl salicylate, and α-pinene, **Table 1**) were attractive to *C. pyri* females (binomial test, p<0.012 and p<0.003, respectively, **Figures 5**, **6**). Over the responsive insects, Mx1 attracted 71% and Mx2 76% of the females tested. No single compounds of the Mx1 were attractive to *C. pyri* females. The Mx6 mixture of (Z)-3-hexenyl acetate and (Z)-3-hexenol had no behavior-modifying activity. However, when the synthetic blend, Mx1, was offered against the pear’s natural odors, there was no significant difference between female’s choices; 56.8% of females chose Mx1 and 43.2% pear plants (p>0.05, **Figure 5**). Motivation of *C. pyri* ranged between 63% and 98%. The behavior of *C. pyrisuga* in response to Mx1 was also observed, and 70% of the females were significantly attracted to the synthetic blend compared to the control solvent (binomial test, p<0.0000, **Figure 5**). However, the motivation of *C. pyrisuga* in the experiment was low at 43%.

**Figure 7:**
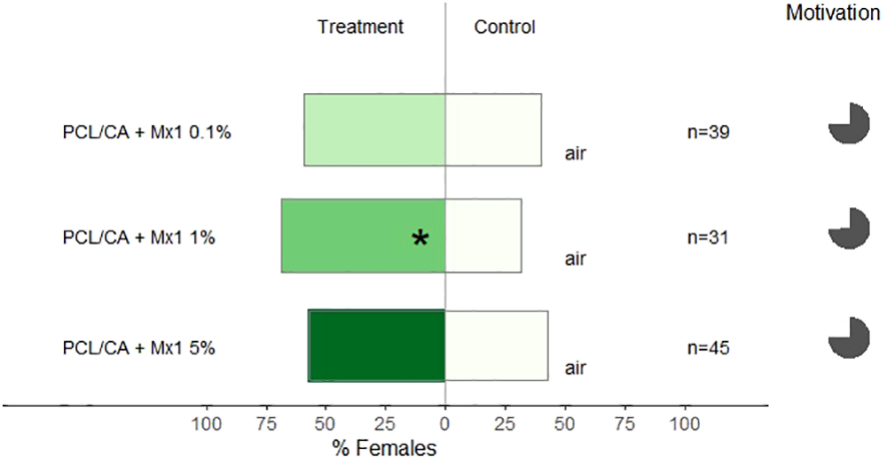
Results of Y-olfactometer-bioassays testing PCL/CA nanoformulations of Mx1 in different concentrations (0.1, 1 and 5%) with females of *Cacopsylla pyri* against pure air. The letter “n” corresponds to the numbers of insects used for the tests. Results are shown as the percentage of psyllids in each arm. Statistically significant differences are indicated with *p < 0.05 (binomial test). The percentage of psyllids that made a choice (dark gray) or not (light gray) is presented as pie charts on the right (“motivation”).

Different concentrations of Mx1 were formulated onto nanofibers, and we observed that only the one with 1% of Mx1 was attractive to females *C. pyri* (binomial test, p< 0.02, **Figure 7**). The increased concentration of the Mx1 to 5% in nanofibers did not lead to more significant attraction (p>0.05, **Figure 7**).

#### 3.2.2 Repellents essential oils

**Figure 8:**
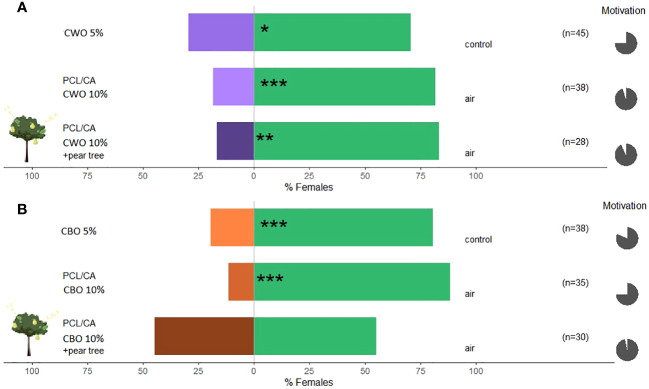
Results of Y-olfactometer-bioassays testing essential oils with females of *Cacopsylla pyri.* **(A)** Cedar wood oil (CWO); **(B)** Cinnamon bark oil (CBO). The letter “n” corresponds to the numbers of insects used for the tests. Results are shown as the percentage of psyllids in each arm. Statistically significant differences are indicated with *p <0.05; **p < 0.01; ***p <0.001 (binomial test). The percentage of psyllids that made a choice (dark gray) or not (light gray) is presented as pie charts on the right (“motivation”).

*C. pyri* females were significantly repelled by CBO 80.6% and CWO oil 70.6% (binomial test, p<0.001 and 0.024, respectively, **Figures 8A, B**) at concentrations of 5% of essential oil. In a subsequent experiment, female *C. pyri* preferred the control arm, avoiding the treatment arm containing the nano-formulated essential oils CWO and CBO at 10% concentration (binomial test, p<0.001 and p<0.0001, respectively, **Figures 8A, B**). CBO nanofibers elicited a slightly higher repellence than CWO nanofibers (88.5% and 81.6%, respectively). A significant host odor masking effect was observed when nano-formulated CWO was offered together with pear plants (binomial test, p<0.01, **Figure 8A**); 83.3% of the responsive females of *C. pyri* could not recognize the odors from pear plants. On the other hand, nano-formulated CBO failed to chase females away from the host plant. 44.8% of the females entered the olfactometer arm, where the natural volatiles of the pear trees and the formulated CBO were offered (**Figure 8B**). The motivation of *C. pyri* was higher than 75.5% in all the performed tests.

### 3.3 Binary−choice oviposition bioassay

**Figure 9:**
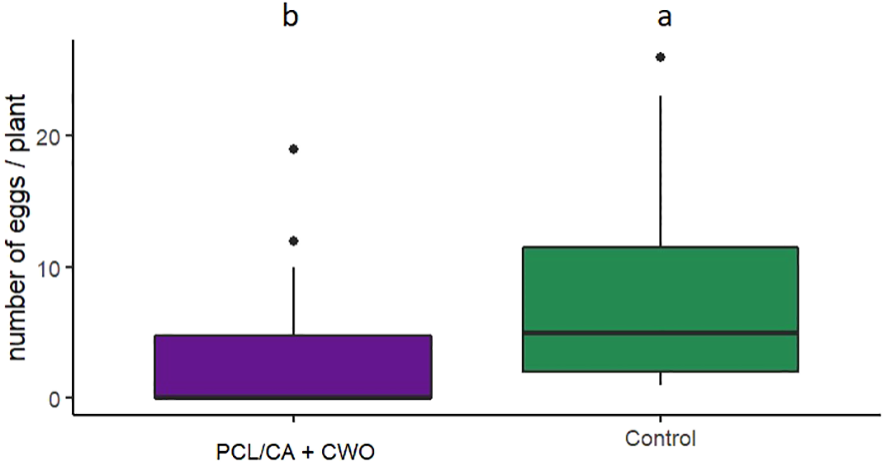
Number of eggs laid by *Cacopsylla pyri* in binary−choice oviposition bioassay on pear branches treated with PCL/CA CWO 10% nanofibers dispensers or non-treated pear branches (n = 20). Boxes correspond to the 25th and 75th percentiles, medians are shown as lines, and whiskers extend to 1.5 times of the interquartile ranges. Different letters indicate statistical differences.

In oviposition binary-choice bioassays, *C. pyri* females laid from none to a maximum of 18 eggs per leaf during the experimental runtime of 72 h. Females laid significantly more eggs on control leaves of pear trees (70.55 ± 10.4%) than on leaves with CWO nanofiber dispensers (29,45 ± 6.2%) (GLM, F_1,169 =_ 4.2621, p=0.03897, **Figure 9**).

### 3.4 Weight loss investigations of dispensers

**Figure 10:**
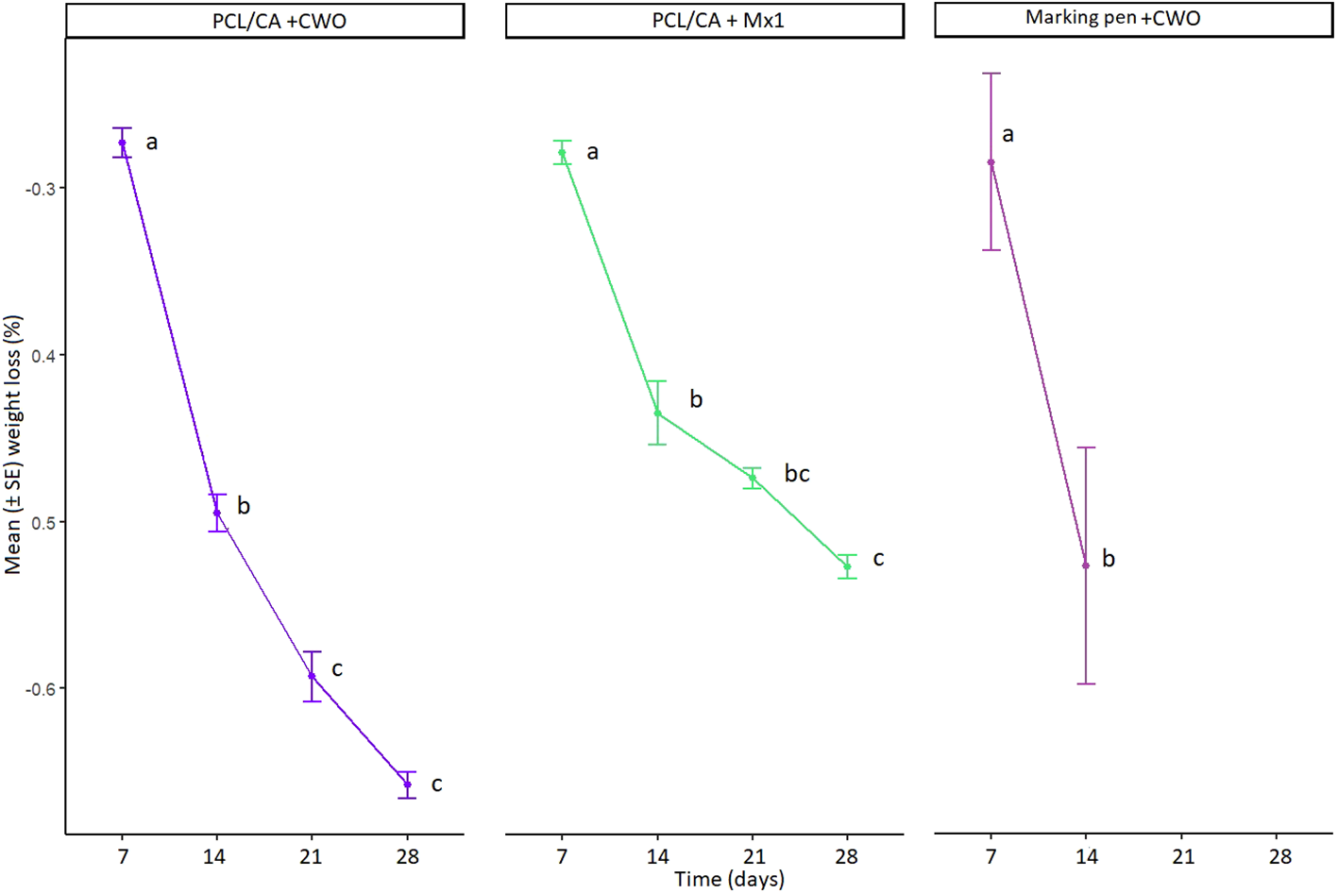
Cumulative percentage of weight loss of PCL/CA nanofibers incorporated with CWO 10% (n=3) and Mx1 (n=3) showing differences in weight loss over time under controlled conditions in laboratory (left and middle graph). Cumulative percentage of weight loss of marking pen dispensers loaded with 10 mL of CWO (n=5) showing difference in weight loss over time under field condition in May 2023 (right graph). The same letters (GLM, Tukey test, p < 0.05) indicate no significant differences.

The profiles of CWO and Mx1 release from the electrospun PCL/CA are shown in **Figure 10**. Clearly, the release profile can be divided into two sections: one representing the highest release after seven days and the other showing the slow release from 14 to 28 days. While the release rate was very low in the samples, it significantly decreased after 14 days up to 28 days (GLM, F_2,24_= 3.9022, p= 0.01403, **Figure 10**). This is due to the low degradability of PCL/CA nanofibers compared to that observed in natural polymers. On the other hand, volatiles release from PCL/CA nanofibers takes place because of its diffusion in the mat, which is a function of the delayed and slow diffusion of the volatiles. These properties make PCL/CA nanofiber mats especially useful as volatile dispensers. The mean percentage weight loss of marking pens was comparable to the CWO nanofibers at 7 (0.285 and 0.273, respectively) and 14 days (0.527 and 0.495, respectively).

### 3.5 Field trials

#### 3.5.1 Trial 1: nanofibers dispensers inside traps

**Figure 11:**
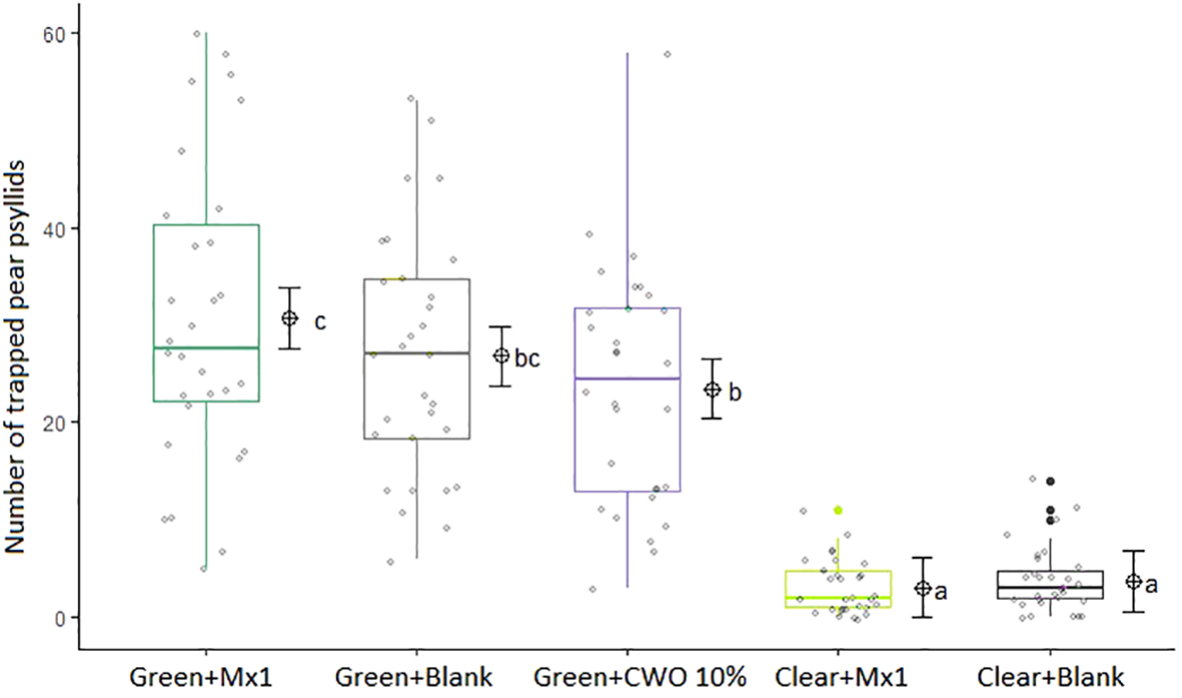
Statistical analysis of *Cacopsylla pyri* adult captures per trap-type: green traps equipped with Mx1-loaded nanofibers, green traps with blank nanofibers or green traps with CWO-loaded nanofibers, clear traps with Mx1-loaded nanofibers or clear traps with blank nanofibers (n=5), in field experiment from May to July 2021 (over 6-week). The same letters (GLM, Tukey test, p < 0.05) indicate no significant differences. Boxes correspond to the 25th and 75th percentiles, medians appear as lines, and whiskers extend to 1.5 times of the interquartile ranges. Grey dots represent raw values and black dots outliers. Corresponding means and confidence intervals predicted from model are shown to the right of each box.

During the six weeks of the field trial, more *C. pyri* adults were captured in green traps baited with nanofibers formulated with the attractive Mx1 blend mimicking the volatile profile from post-flowering pear foliage than in green traps with nanofibers with CWO (GLM, F_5,140_= 112.2, p=0.024, **Figure 11**). However, green traps baited with blank nanofibers captured as many *C. pyri* adults as green traps with CWO or Mx1 nanofibers. Transparent traps caught fewer insects than all green traps (p>0,001), and there were no statistical differences between insect captures on clear traps baited with Mx1 and blank nanofibers.

#### 3.5.2 Trial 2: marking pen as repellent dispenser

**Figure 12:**
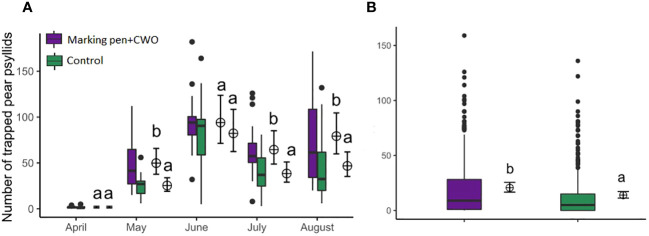
Number of pear psyllids trapped on green traps located at the border of areas either treated with marking pen dispensers filled with CWO (violet box) or untreated (control, green box) in a pear orchard (number of traps=20). Experiment performed from April to August 2023. **(A)** Comparisons of monthly captures between the areas; **(B)** Comparison between areas over the complete period of the field trial. The same letters (GLM, Tukey test, p < 0.05) indicate no significant differences. Boxes correspond to the 25th and 75th percentiles, medians appear as lines, and whiskers extend to 1.5 times of the interquartile ranges. Black dots represent outliers. Corresponding means and confidence intervals predicted from model are shown to the right of each box.

In the second field trial, an increased psyllid capture of pear psyllids was observed in traps mounted along the perimeter in the area treated with marking pen dispensers filled with CWO compared to traps on the perimeter of untreated (control) area (GLM, F_1,194_= 24.448, p < 0.001, **Figure 12A**). There were also differences in psyllids captures per trap between marking pens CWO treated and the untreated area over the evaluated months (GLM, F_4,195_= 141.727, p < 0.001, **Figure 12B**). Significantly more psyllids were captured in the CWO-treated area in May, July, and August (GLM, F_4,195_= 141.727, p= 0.0002; p=0.0035; p=0.0027, respectively).

## 4 Discussion

Many herbivorous insects use specific blends of plant-emitted volatiles to identify and locate suitable host plants (Bruce and Pickett, 2011). In insect-plant interactions, a similar specificity might originate from maintaining a particular ratio in the plant-released volatile blends (Bruce et al., 2005). Insect attraction disappeared when the ratios of the essential compounds, as identified in the headspace of the host plant, were replaced by the proportions of the same compounds emitted by a non-host plant (Tasin et al., 2006). Similarly, responses of olfactory receptor neurons seem to indicate that host plant discrimination by herbivore insects must be mediated by the ratio of the compounds in the volatile blend (Bichão et al., 2003). The blend Mx1 consisting of two esters ((Z)-3-hexen-1-yl acetate, methyl salicylate), three terpenoids (α-pinene, (*Z*)-ocimene, (*E*)-ocimene), and one alcohol (*Z*)-3-hexen-1-ol), was attractive in olfactometer assays to both psyllid species *C. pyri* and *C. pyrisuga*. Furthermore, the Mx 2 consisting of methyl salicylate, α-pinene, (*Z*)-ocimene, and (*E*)-ocimene was attractive to *C. pyri*. To the best of our knowledge, there are to date no specific studies on the reaction of *C. pyrisuga* to plant volatiles, and in the present study, we could demonstrate for the first time that this species reacts to host volatiles. Unfortunately, the limited number of insects of this species available did not allowed to perform further trials with *C. pyrisuga*. The blend Mx6 containing only green leaf volatiles (GLVs) did not trigger attraction in behavior tests despite being detected by *C. pyri* antennae in EAG tests. The reason behind the ability of *C. pyri* to detect such GLVs remains unclear. However, GLVs are important in host plant location in other psyllid species (Moran and Brown, 1973; Lapis and Borden, 1993; Soroker et al., 2004; Horton and Landolt, 2007). Wenninger et al. (2009) showed that Asian citrus psyllid responded to foliar volatiles of several host plants in a Y-olfactometer. They also showed that olfactory cues enhanced the psyllid’s response to visual cues, indicating that Asian citrus psyllid can integrate information from at least two sensory modalities during host plant search. The ability to correlate odor with color cues may facilitate a phytophagous insect’s ability to recognize the overall stimuli signature from its host plant and differentiate it from surrounding vegetation (Patt and Setamou, 2010).

All five compounds evaluated in this work were perceptible by *C. pyri* in EAG experiments. Mx1, the most complex blend, triggered antennal reactions in both psyllid species (C. *pyri* and *C. pyrisuga*). However, few data are available on the morphological structure of the olfactory system of psyllids, which could further explain the importance of such volatiles on host location (Gross et al., 2021). Scanning electron micrographs of the antennae of insects of the taxa Psyllidae, Cicadellidae, and Fulgoromorpha revealed few olfactory sensillas (Kristoffersen et al., 2006; Kristoffersen et al., 2008; Onagbola et al., 2008; Arras et al., 2012; Coutinho-Abreu et al., 2014; Zheng et al., 2020; Gross et al., 2021). There are only five olfactory sensilla in each of the antennae of Asian citrus psyllid (*Diaphorina citri*) (Nagata et al., 2003; Onagbola et al., 2008; Arras et al., 2012; Zheng et al., 2020). This suggests that the perceptibility of odorants by *C. pyrisuga* and *C. pyri* could be limited by a possible small number of sensilla. More detailed research on the morphology of antennae of these species, as well as single sensilla recordings, may help to uncover more details about odors recognition. Carrot psyllid (*Trioza apicalis*) is known to possess around 50 olfactory receptor neurons (ORNs) (Kristoffersen et al., 2006). This number is not nearly as large as for the fruit fly *Drosophila melanogaster*, which has about 1200 ORNs per antenna (Stocker, 1994), so psyllids are thought to have a much more specialized olfactory system (Kristoffersen et al., 2008). Other studies with psyllids have demonstrated host-finding preferences based on olfactory stimuli. For example, Soroker et al., 2004 showed that females of *Cacopsylla bidens* were attracted to uninfested pear trees, and plants colonized with other adults or with nymphs were less preferred. Similarly*, C. picta* females prefer apple trees that are not infected with phytoplasmas for oviposition (Mayer et al., 2011; Görg et al., 2021). In addition, electroantennographic studies demonstrated a significantly stronger antennal response of females of *C. pyri* to extracts containing pear leaf scents than to the aerial control. Thus, it can be assumed that plant odors are perceptible for psyllids in general and at least psyllid females are attracted to the odors of their respective host plant. A current study showed that altered volatile emission of pear trees under elevated atmospheric CO_2_ levels is irrelevant to pear psyllid host choices. Changes in the relative release of specific compounds such as hexanal and γ-terpinene from pear odors did not impact the behavior of the highly specialized pest insect *C. pyri* (Gallinger et al., 2023). The perceptibility of α-pinene was also demonstrated for another migratory species, *C. pruni*. α-pinene is a prominent component of the scent profile of *Abies alba* (Gallinger et al., 2020), a potential overwintering host for several migratory psyllid species (Hodkinson, 2009; Thébaud et al., 2009; Jarausch and Jarausch, 2016). Due to the complexity of the life cycle of host-shifting species, such species are expected to perceive scents typical for their reproduction and overwintering host plants’ particular components, and utilize them for host-finding. This is also shown by the study of Kristoffersen et al., 2008 (Kristoffersen et al., 2008) investigating the migrating psyllid species *T. apicalis*, which showed significant electrophysiological responses to scent extracts of carrot plants, spruce, pine, and juniper. Since *C. pyri* does not change its host plant, the perceptibility of fewer odorants compared to *C. pyrisuga* is not unlikely. In particular, significant antennal signals were triggered by cis-3-hexenyl acetate perceived by the *Prunus* dwelling psyllid *C. pruni* (Gallinger et al., 2020).

Essential oils have demonstrated effectiveness in repelling various insect species, with notable success in combating blood-feeding pests that pose a threat to humans, such as mosquitoes (Sukumar et al., 1991; Carroll and Loye, 2006; Grison et al., 2020; Chatterjee et al., 2023). Additionally, essential oils have proven to be efficient in safeguarding stored-product crops (Karabörklü and Ayvaz, 2023). The prevailing belief is that these oils disrupt insect orientation by masking host odors, potentially incorporating an additional element of direct toxicity (Regnault-Roger, 1997; Regnault-Roger et al., 2012). Botanical oils, known for their safety on food crops and acceptance in organic farming (Ribeiro et al., 2016), present versatile options for rotation with traditional insecticides. Specific non-host botanical oils, including cedarwood and thyme oils, have been identified to reduce *D. citri* infestation in citrus for short periods following application (24 h) (Regnault-Roger et al., 2012). Our findings confirmed that CWO reduced the response of *C. pyri* to pear volatiles in the laboratory in olfactometer tests, oviposition trials, and field trials in this study.

In field trial 1, colored green traps baited with Mx1 nanofibers were more attractive than green traps baited with CWO nanofibers, but no significant differences were observed when comparing captures to the control green traps equipped with blank nanofibers only. Other studies showed that adding dispensers loaded with VOCs to traps did not enhance the insects’ attraction. The possible explanation is that under field conditions, there is likely competition between the synthetic volatiles emitted from a lure and those released from other plants in the background (Martini et al., 2020). These volatiles in the field may compete or otherwise inhibit the attraction of the target insect to the synthetic point sources. Therefore, behavioral responses obtained in olfactometer assays may differ from those observed in the field (Gregg et al., 2018). In our olfactometer trial, when *C. pyri* was confronted with odors emitted from nanofiber loaded with Mx1 and a pear plant, they were equally attracted to both odor sources. In another investigation, plant volatile mixtures did not increase the attraction of *Halyomorpha halys* adults to baited pyramid traps but added a slight increase in the retention of individuals (Morrison et al., 2018). In this study, every plant volatile mixture contained several GLVs, some of which were shared between mixtures, suggesting that GLVs may play an important role in foraging decisions by *H. halys* adults.

In our study, we selected only the complex blends Mx1 for field trial, even though both Mx1 and Mx2 blends yielded comparable behavioral results in the laboratory olfactometer tests. We decided this, based on previous results, as the increased attraction of *D. citri* to yellow sticky traps has only achieved with the most complex bend (Martini et al., 2020). These outcomes suggest that *C. pyri* may use several general volatiles associated with pear cultivars during host finding rather than a specific olfactory cue, or olfactory cues could play a secondary role in pear psyllids foraging, and other sensory modalities like visual signals are more relevant for host finding. In other research, the relevance of the visual factor has already been explored for *C. pyri*, showing that specific wavelengths (green color) are attractive to this species (Czarnobai De Jorge et al., 2022; Czarnobai De Jorge et al., 2023). Another possibility could be that *C. pyri* may exhibit phenotypic plasticity concerning olfactory responses to hosts, allowing the recognition of different volatile blends associated with different *Pyrus communis* cultivars. Further investigations are necessary to investigate a more specific kairomone for *C. pyri*.

The development of appropriate dispensers are a crucial component for the success of the use of VOCs under field conditions. EOs have already been incorporated into nanofibers for pest control in agriculture, but fewer works have reported using this technology for insect management (Czarnobai de Jorge and Gross, 2021). For example, polyethylene glycol–coated nanoparticles loaded with garlic essential oil are effective against red flour beetle [*Tribolium castaneum* Herbst. (Coleoptera: Tenebrionidae)] (Yang et al., 2009). The coating provided by the polymer surrounding the EO molecules can prevent them from being exposed to environmental conditions, extending their shelf life and stabilizing these products for longer periods (Anjali et al., 2012). Recently, nanofibers produced with a PCL/CA polymer blend extended the release rates of eugenol (clove essential oil major compound) for more than nine weeks. However, in the field, they did not reduce the capture of *C. pyri* to attractive green sticky traps as expected (Czarnobai de Jorge et al., 2022).

In our second field test, using marking pens as CWO dispensers in a different experimental design, significantly more psyllids were captured on attractive green sticky traps at the margin of the treated compared to untreated area. We hypothesize that *C. pyri* could be scattered by the repellent in this area and tried to relocate, moving away from the repellent-treated area. Further experiments are necessary to prove this hypothesis. A randomized block design with more repetitions and beating the limb of pear trees on repellent-treated plots, together with counts of eggs and nymphs on branches/leaves, could yield more detailed data for further investigation on the efficacy of repellents in field systems. In the binary choice oviposition bioassay, an oviposition reduction was observed on trees treated with CWO nanofibers under lab conditions. Therefore, in subsequent field trials similar results can be expected. However, in the analysis of the data from both field trials, there was no discrimination between the catches of males and females in the traps, so the field data may have been influenced by a different behavior of the males.

A similar approach investigating the use of fir oil as repellent *against D. citri* was investigated recently. In this study, the authors assessed a reduction in the number of insects collected in areas treated with glycerin dispensers containing fir oil (Chuang et al., 2023). Research on *H. halys* by Zhang et al., 2014 found that plant essential oil blends (clove, lemongrass, and spearmint oil), as well as individual plant volatiles from those essential oils (e.g. l-carvone, eugenol, trans/cis-citral, methyl benzoate, pulegone, methyl salicylate, β-caryophyllene, among others), resulted in inhibition of otherwise attractive traps (Zhang et al., 2014). It could also be possible that blends of known repellent essential oils such as the ones tested in the present work and clove oil (Czarnobai De Jorge et al., 2022) could increase the repellency of pear psyllids in the field. Furthermore, a detailed investigation on the specific individual compound of essential oils of CBO, CWO, and clove (e.g., eugenol) could also have a higher repellents effect against pear psyllids, which can be investigated in future studies. Nanofibers have been demonstrated to extend the release rates of volatiles for several weeks, but their use and further development is still limited by longer time consuming of production with lab equipment and limitations on the maximum load of the active compound on the fibers matrix. Our results suggest that further investigation of EOs as repellents against pear psyllids in combination with attractants for push-pull is warranted.

## 5 Conclusion

In conclusion, this research has provided a comprehensive exploration of the dynamics of herbivorous insect behavior in response to plant-emitted volatiles, particularly in the context of pear psyllids. Through a combination of laboratory experiments and field trials, we have elucidated some of the intricate relationship between volatile blends and insect attraction, as well as the potential efficacy of essential oils as repellents. Additionally, our research highlights the promising role of botanical oils, such as cedarwood oil (CWO), in disrupting insect orientation and potentially deterring them from pear plants.

The deployment of push-and-pull strategies, consisting of both attractive volatile blends and repellents, presents a multifaceted approach to insect pest management. While field trials have shown varying results, indicating the need for further optimization, the overall potential of these strategies is promising for integrated pest management practices. The use of repellents in the field can drive away males and females, thus reducing their encounter and mating, resulting in reduction of female’s oviposition. Furthermore, the results of field trial 2 might indicate that also green attractive traps have a pull effect, which can be combined with repellents for a push-and-pull control method. Moreover, the use of innovative delivery tools, such as nanofibers and marking pens as dispensers for the emission of volatile compounds, presents opportunities to enhance the efficacy and longevity of VOCs for pest control interventions. For the management of pear decline, implementing a push-and-pull strategy at the beginning of the spring could reduce the re-entry of psyllids into orchards and prevent new infections by overwintering insects, leading to a reduction in the population of psyllids, resulting in lower rates of phytoplasma infection.

This study contributes valuable insights into the complex interplay between plants and herbivorous insects, providing a basis for the development of more sustainable and effective pest management strategies. Continued research efforts in this area will be crucial for mitigating pest pressures and reducing phytoplasma infection enhancing agricultural productivity in the face of evolving environmental challenges.

## Data availability statement

The datasets presented in this study can be found in online repositories. The names of the repository/repositories and accession number(s) can be found in the article/supplementary material.

## Ethics statement

The manuscript presents research on animals that do not require ethical approval for their study.

## Author contributions

BC: Conceptualization, Data curation, Formal analysis, Funding acquisition, Investigation, Methodology, Software, Validation, Visualization, Writing – original draft, Writing – review & editing. AK: Data curation, Formal analysis, Investigation, Writing – review & editing. HH: Writing – review & editing. JG: Conceptualization, Methodology, Project administration, Resources, Supervision, Writing – review & editing.
